# Social Use of Facial Expressions in Hylobatids

**DOI:** 10.1371/journal.pone.0151733

**Published:** 2016-03-15

**Authors:** Linda Scheider, Bridget M. Waller, Leonardo Oña, Anne M. Burrows, Katja Liebal

**Affiliations:** 1 Dept. of Education and Psychology, Freie Universität Berlin, Berlin, Germany; 2 Dept. of Psychology, University of Portsmouth, Portsmouth, United Kingdom; 3 Dept. of Natural Science and Mathematics, University of Groningen, Groningen, The Netherlands; 4 Dept. of Physical Therapy, Duquesne University, Pittsburgh, Pennsylvania, United States of America; 5 Dept. of Anthropology, University of Pittsburgh, Pittsburgh, Pennsylvania, United States of America; University of Veterinary Medicine Hannover, GERMANY

## Abstract

Non-human primates use various communicative means in interactions with others. While primate gestures are commonly considered to be intentionally and flexibly used signals, facial expressions are often referred to as inflexible, automatic expressions of affective internal states. To explore whether and how non-human primates use facial expressions in specific communicative interactions, we studied five species of small apes (gibbons) by employing a newly established Facial Action Coding System for hylobatid species (GibbonFACS). We found that, despite individuals often being in close proximity to each other, in social (as opposed to non-social contexts) the duration of facial expressions was significantly longer when gibbons were facing another individual compared to non-facing situations. Social contexts included grooming, agonistic interactions and play, whereas non-social contexts included resting and self-grooming. Additionally, gibbons used facial expressions while facing another individual more often in social contexts than non-social contexts where facial expressions were produced regardless of the attentional state of the partner. Also, facial expressions were more likely ‘responded to’ by the partner’s facial expressions when facing another individual than non-facing. Taken together, our results indicate that gibbons use their facial expressions differentially depending on the social context and are able to use them in a directed way in communicative interactions with other conspecifics.

## Introduction

In searching for the evolutionary roots of human communication, comparative researchers have dedicated much attention to the question whether communication of non-human primates is also characterized by voluntary and intentional use of different signal types, which is a key feature of human communication. In which case, non-human primates should use their signals purposefully, direct them to other group members and adjust them to the attentional state of the recipient. This would indicate that they have some voluntary control over the production of their signals. There are only a few studies investigating systematically whether and how non-human primates use facial expressions in social interactions. Waller et al. [1] found that orang-utans modify their facial expressions depending on the attentional state of the recipients. The authors offer a lower-level explanation for the differential use of facial expressions, since sensitivity to the attentional state of others could be the result of the salience of the face as a social stimulus. However, it is important to emphasize that currently there is only a small set of studies available that provide data on the use of facial expressions as a function of the recipient’s attentional state.

Most existing research on facial expressions has been devoted to investigating the role of facial expressions for coordinating social interaction, facilitating group cohesion and the maintenance of individual social relationships in non-human primates [2–6], including several species of monkeys and great apes (e.g., *Pan troglodytes*: [7–9]; *Pongo pygmaeus*: [10–13]; *Macaca mulatta*: [14–17]; *Callithrix jacchus*: [18–22]), but little is known about the various species of small apes (Hylobatidae) [23–25]. Gibbons are equipped with extensive facial muscles [26], which they use to perform a variety of facial movements [25,27]. Still, from the little that is currently known, their facial communication seems less complex than those of more terrestrial and/or socially more complex primate species [24,26].

The newly developed Facial Action Coding System for hylobatid species (GibbonFACS: [24]) offers the opportunity to examine facial expressions in much greater detail than previous methods and enables the objective and standardized comparison of facial movements across species [28,29]. The coding system can be used to identify the muscular movements underlying facial expressions and thus used to define facial expressions as a combination of such facial muscle contractions (Action Units [AUs]) or more general head/eye movements (Action Descriptors [ADs]). In a previous study we used GibbonFACS and provided a detailed description of the repertoire, the rate of occurrence and the diversity of facial expressions in five different hylobatid species comprising three genera (*Symphalangus*, *Hylobates and Nomascus*) [25]. The focus of the investigation was to compare the different genera and to reveal potential correlations with socio-ecological factors (group size and monogamy level) on a species level. In the current study we focus on how hylobatids adjust their use of facial expressions to the behaviour of a recipient. Liebal et al. [23] observed four distinct facial expressions in siamangs (‘mouth-open half’, ‘mouth-open full’, ‘grin’, and ‘pull a face’), which were used across different social contexts. Most importantly, these four types of facial expressions were almost exclusively used when the recipient was visually attending. This is currently the only evidence indicating that hylobatids adjust the use their facial expression to the behaviour of their audience.

To confirm these previous findings and to systematically investigate the use of facial expressions in hylobatids in social contexts, we explored the influence of the attentional state of a potential receiver on the production of facial expressions. Additionally, in order to examine the influence of a given facial expression on the recipient’s behaviour, we investigated whether recipients respond themselves by using a facial expression. To do so, we analysed the distribution of consecutive facial expressions (two facial expressions between the pair partners within a defined period of time), but excluded identical facial expressions to rule out more basic, reflexive responses like facial mimicry (the repetition of the same facial expression of the sender by the receiver; [13,30,31]).

If facial expressions of hylobatids have a social function and individuals are capable of adjusting their use to the behaviour of the recipient and the context in which they are used, we predicted that 1) senders use facial expressions with longer duration when the recipient is facing the sender than when they are not and 2) senders use facial expressions more frequently in social than in non-social contexts when the recipient is visually attending. Both this longer duration and the more frequent use of facial expressions in social contexts would enhance signal transmission and indicate that they indeed have an intended communicative function instead of merely representing undirected behaviours. Furthermore we predicted that 3) consecutive facial expressions, which indicate that the recipient responds to the sender’s facial expression by producing another facial expression, are more common when individuals are facing each other than when they are not.

## Materials and Methods

### Ethics Statement

Animal husbandry and research comply with the ‘‘EAZA Minimum Standards for the Accommodation and Care of Animals in Zoos and Aquaria”, the ‘‘WAZA Ethical Guidelines for the Conduct of Research on Animals by Zoos and Aquariums” and the ‘‘Guidelines for the Treatment of Animals in Behavioral Research and Teaching” of the Association for the Study of Animal Behavior (ASAB). IRB approval was not necessary because no special permission for the use of animals in observational studies is required in Germany. Further information on this legislature can be found in paragraphs 7.1, 7.2 and 8.1 of the German Protection of Animals Act (‘‘Deutsches Tierschutzgesetz”). Our study was approved by all participating Zoos.

### Subjects

Mated pairs of five different gibbon species were observed, comprising three pairs of siamangs (*Symphalangus syndactylus)*, two pairs of pileated gibbons (*Hylobates pileatus*), one pair of white-handed gibbon (*Hylobates lar*), one pair of yellow-cheeked gibbon (*Nomascus gabriellae*) and one pair of southern white-cheeked gibbon (*Nomascus siki*), resulting in a total of 16 individuals. All pairs except one were housed together with their one to three offspring (for details of the individuals and group composition see S1 Table).

### Data collection and coding

Data collection took place between March 2009 and July 2012 in different zoos in the UK (Twycross), France (Mulhouse), Switzerland (Zurich) and Germany (Rheine). The behaviour of each pair was video-recorded in 15 min bouts using the focal animal sampling method [32] (with both animals always in view). We focused on situations when the pair was in reaching distance and so had the opportunity to interact and we coded only facial expressions that occurred when being in that distance to each other. Recordings were evenly distributed across different times of the day and conducted on several different consecutive days. This resulted in in a total of 21 hours of observation, a mean observation time of 158 minutes per pair (SD = 34 min). We later coded the video footage with the software Interact (Mangold International GmbH, Version 9.6) and identified facial expressions using GibbonFACS [24]. A facial expression was defined using GibbonFACS, as a single or a combination of more than one facial movement (so-called Action Units (AU) or Action Descriptors (AD)), regardless whether it was used in interactions with others or not. The facial expression was coded at the apex of the expression. In total, 1080 facial expression events were identified. For each facial expression, we also measured its duration (from the onset to apex to offset) and coded whether individuals were facing each other while producing the facial expression (if the faces of both individuals were clearly directed at each other). All other instances were coded as non-facing. For each facial expression, we also coded the context in which it was used. We differentiated between social contexts (Agonism, Play, Grooming) and non-social contexts (Selfgrooming, Resting) and coded “Unclear” when we were not able to assign any of those contexts. We conducted a reliability analysis on 10% of the data, which was calculated using Wexler’s Agreement as for the human FACS and all other non-human animal FACS systems [33]. Agreement was 0.83, which in FACS methodology is considered very good agreement [33]. Agreement for the measurements of context (social, non-social, unclear) and facing (facing vs non-facing) were tested using Cohen’s Kappa [34] and were substantially to almost perfect (context: *K* = 0.74, *P* < 0.001; facing: *K* = 0.85, *P* < 0.001).

### Statistical Analyses: Duration of facial expressions

To test whether the duration of facial expressions (response variable; log-transformed) was influenced by individuals of a mated pair facing each other (thereafter 'facing') or not and/or whether they used facial expressions in a social context compared to non-social (thereafter 'context') we used a Linear Mixed Effects Model (LMM; [35]) into which we included these two predictors as well as their interaction as fixed effects, and individual and number of video clip as random effects. To keep type I error rates at the nominal level of 5%, we included random slopes of facing and context (manually dummy-coded) and their interaction within individuals and video clip, but not the correlation parameters between the predictors and nor the random intercept [36,37]. We used R [38] and lme4 [39] to perform the LMM.

We checked for model stability by excluding levels of the random effects one at a time from the data and comparing the estimates derived with those obtained from using the entire data set which indicated no influential cases to exist. Variance inflation Factors (VIF; [40]) were derived using the function vif of the R-package car [41] applied to a standard linear model with random effects and the interaction excluded and found that collinearity was not an issue (maximum VIF: 1.14). We checked whether the assumptions of normally distributed and homogeneous residuals were fulfilled by visual inspection of a qq-plot of the residuals and residuals plotted against fitted values, which indicated no violation of these assumptions.

An overall test of the significance of the fixed effects as a whole [36] was obtained using a likelihood ratio test (R function anova with argument test set to “Chisq”) testing the full model against the null model (comprising only the random effects). P-values for the individual effects were based on likelihood ratio tests comparing the full with the respective reduced models (R function drop1; [37]).

## Results

### Types of facial expressions

In total, we found 45 different types of facial expressions (see S2 Table). Some types were single AUs or ADs; however, the majority were combinations of several AUs and/or ADs. Most types of the facial expressions were used in both facing and non-facing situations (24 types) followed by types, which were used only in non-facing situations (17) and only 4 types were exclusively used in facing situations (see Table 1).

**Table 1:** Type of facial expressions used exclusively when facing another individual, those used exclusively when not facing another individual, and those that occurred in both facing and non-facing situations. For more details on the morphology regarding previous descriptions of these facial expressions, see S2 Table. All facial expressions including AU10, AU16, AU25, AU26, AU27 are forms of ‘open-mouth’ displays [23]. Frequencies and other details are reported in S2 and S3 Tables.

Use of facial expressions (number of types of facial expressions)	Facial expressions (single AUs/Ads or in combination with other AUs/Ads)
**Only Facing (4)**	{AU9+AU10+AU16+AU25+AU27} {AU10+AU12+AU25+AU27} {AU16+AU25+AU26+AUEye*} {AU1+2+AU10+AU16+AU25+AU27}
**Only Non-Facing (17)**	{AU1+2} {AU8} {AU12} {AU17} {AD500} {AU1+2+AU18} {AU10+AU25} {AU16+AU25} {AU41+AUEye*} {AU7+AU25+AU26} {AU1+2+AU5+AU25+AU26} {AU8+AU25+AU26+AD19} {AU9+AU10+AU25+AU27} {AU12+AU25+AU26+AD37} {AU25+AU26+AUEye+AD37} {AU25+AU26+AD37+AD500} {AU10+AU12+AU16+AU25+AU27+AUEye*}
**Facing and Non-Facing (24)**	{AU18} {AU25} {AU41} {AUEye*} {AU25+AU26} {AU25+AU27} {AU8+AU25+AU26} {AU10+AU25+AU26} {AU10+AU25+AU27} {AU12+AU25+AU26} {AU12+AU25+AU27} {AU16+AU25+AU26} {AU16+AU25+AU27} {AU18+AU25+AU26} {AU25+AU26+AD19} {AU25+AU27+AD19} {AU25+AU26+AD37} {AU8+AU25+AU26+AD37} {AU10+AU16+AU25+AU26} {AU10+AU16+AU25+AU27} {AU12+AU16+AU25+AU26} {AU12+AU16+AU25+AU27} {AU10+AU12+AU16+AU25+AU26} {AU10+AU12+AU16+AU25+AU27}

(*AUEye includes either AU43 (eye closure) or AU45 (eye blink), we did not differentiate between the two AUs here).

### Duration of facial expressions when facing another individual compared to non-facing in social versus non-social context

**Fig 1:**
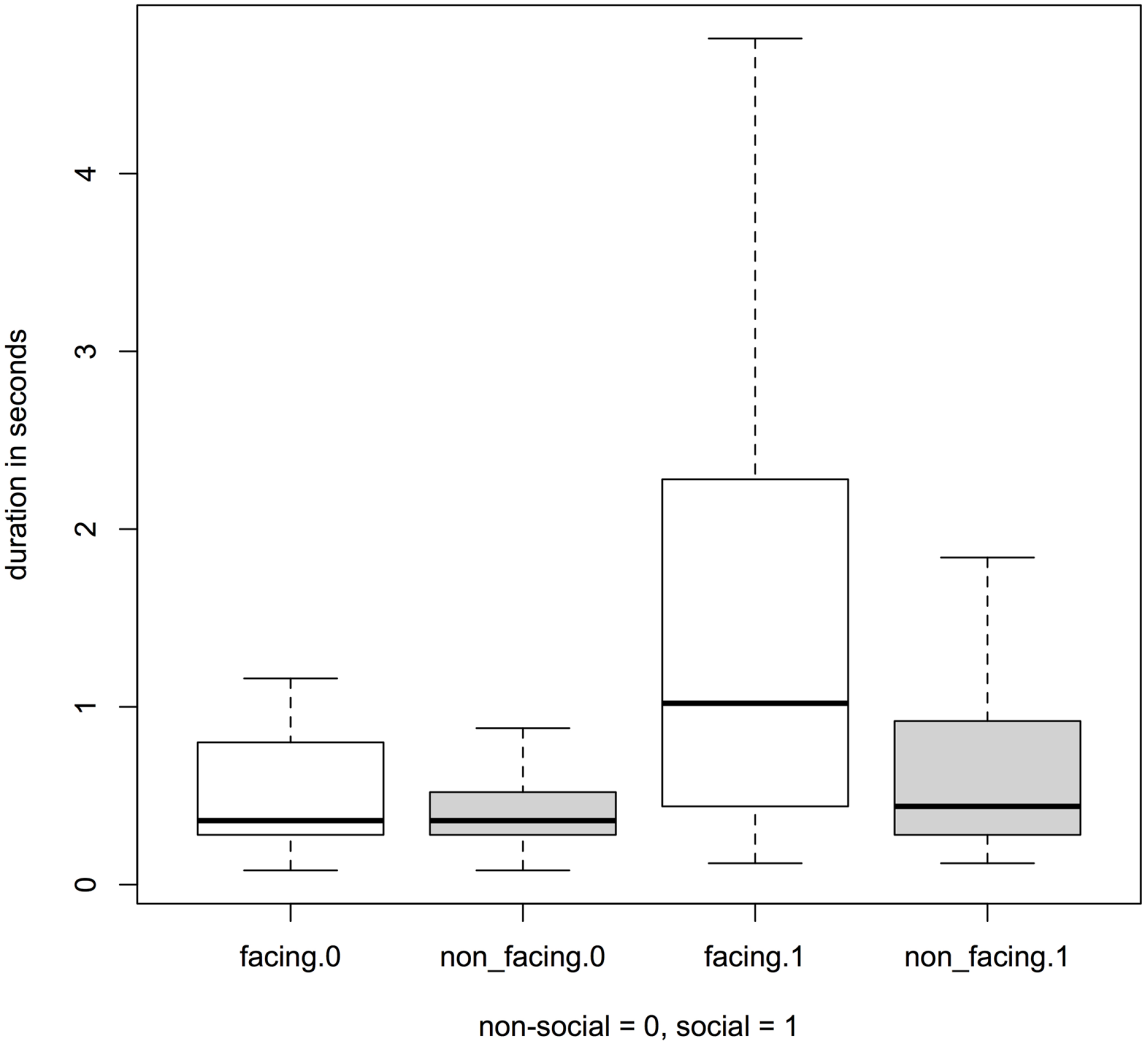
Duration (in sec) of facial expressions when facing another individual compared to not facing in social and non-social contexts. Facial expressions while facing another individual were significantly longer compared to non-facing events, but only in social (right) and not in non-social (left) situations (outliers were excluded for better visualization).

By using a Linear Mixed Effects Model (see Material and Methods for details), we tested the influence on different factors on the duration of facial expressions and found that the full model was significantly different from the null model (likelihood ratio test: *χ*^2^_3_ = 19.96, *P* < 0.001; see also Table 2) indicating that the three fixed effects (facing, context and their interaction) as a collective have an impact on the response. A test of the significance of the interactions was obtained from applying the function drop 1 [37] to the full model. It revealed a significant interaction between facing and context (likelihood ratio test of the interaction: *χ*^2^_1_ = 5.32, *P* = 0.02; Fig 1). Thus, facial expressions produced while facing another individual had a longer duration compared to non-facing events, but only in social and not in non-social contexts (see Estimates and Std. Errors in Table 2; Full Model: Duration).

**Table 2:** Estimates and Std. Errors for the coefficients of the two above-mentioned full models.

Model	Estimates	Std. Error	Lower CL	Upper CL
**Full Model: Duration**				
(Intercept)	6.320	0.145	6.035	6.607
FacingNon-facing	-0.167	0.143	-0.447	0.114
ContextSocial	0.398	0.170	0.047	0.735
FacingNon-facing: ContextSocial	0.398	0.172	-0.736	-0.06
**Full Model: Facing**				
(Intercept)	2.417	0.177	2.083	2.833
ContextSocial	-1.705	0.28	-2.24	-1.115

### Frequency of facing events versus non-facing events in social compared to non-social contexts

To test whether facial expressions while facing another individual (response variable, binary) were used more frequently in social compared to non-social contexts, we used a Generalized Linear Mixed Model with binomial error structure and logit link function [42] into which we included context (predictor) as a fixed effect, and individual and video clip as random effect. We also included the random slopes of context (manually dummy coded) within individual and video clip into the model. Apart from that we used the same software and procedures as described above.

Overall, the full model was significantly different from the null model (likelihood ratio test: *χ*^2^_1_ = 13.84, *P* < 0.001), indicating that the fixed effect had an impact on the response variable. Results showed that the occurrence of facial expressions while facing another individual was more common in the social context compared to the non-social context (see Estimates and Std. Errors in Table 2; Full Model: Facing). Importantly, this difference did not result from a longer duration of facing events compared to non-facing events in social contexts. In social contexts, gibbons did still not face each other in 74,6% of the time.

### Pattern of consecutive facial expressions

In order to test whether the behaviour of the recipient is influenced by the use of a facial expression of the other individual, we compared consecutive facial expressions in pairs when individuals were facing to when they were not facing each other. In order to rule out facial mimicry [13,30,31], we focused on consecutive, but different types of facial expressions. To investigate whether two consecutive facial expressions (response, binary) were more common in situations in which individuals faced each other compared to non-facing situations, we used a Generalized Linear Mixed Model with binomial error structure and logit link function into which we included facing (predictor) as a fixed effect, and individual as random effect. We also included the random slope of facing (manually dummy coded) within individual into the model. Apart from that we used the same software and procedures as described above. Testing for different cut-offs (time intervals between the consecutive facial expressions), we found that the full model was significantly different from the null model (results for different cut-offs see Table 3). Results show that consecutive facial expression events were more common while individuals were facing each other compared to non-facing events (likelihood ratio tests for different cut-offs see Table 3). Results of the analyses including the mimic events showed the same pattern (likelihood ratio tests for different cut-offs see S4 Table).

**Table 3:** Results of likelihood ratio tests of GLMM. Cut-off refers to the time interval between the consecutive facial expressions.

Cut-off	Estimate	Std. Error	Lower CL	Upper CL	*χ*^2^ (df)	*P*-value
**2 seconds**					7.348	6.7 * 10^3^
(Intercept)	-3.288	0.34	-4.047	-2.683		
facingNonfacing	-1.854	0.825	-4.228	-0.475		
**3 seconds**					6.599	10.2 * 10^3^
(Intercept)	-2.987	0.296	-3.884	-2.453		
facingNonfacing	-1.444	0.61	-3.082	-0.345		
**4 seconds**					9.101	2.6 * 10^3^
(Intercept)	-2.752	0.266	-3.718	-2.266		
facingNonfacing	-1.701	0.611	-3.32	-0.578		
**5 seconds**					8.421	3.7 * 10^3^
(Intercept)	-2.618	0.251	-3.452	-2.157		
facingNonfacing	-1.512	0.565	-3.047	-0.49		

## Discussion

In this study, we investigated whether different gibbon species adjusted their usage of facial expressions in interactions with others to the recipients’ behaviour and thus use them differently when others are visually attending versus not attending. Gibbons directed at least some of their facial expressions to specific individuals when they were visually attending and facial expressions that were used when both individuals were facing each other lasted significantly longer than those not directed at others. A similar pattern has been reported for orangutans (*Pongo pygmaeus*) in the context of social play [1]. Interestingly, our study shows that this pattern is present only in social but not in non-social contexts (social context included grooming, agonistic behaviour and play; non-social context included self-grooming and resting). It is important to note that in both social and non-social contexts, individuals were in close proximity to another, but only when in social contexts did facing another individual elicit longer durations of their facial expressions. The same pattern was not observed in non-social contexts. Therefore, we can exclude the possibility that a simple ‘looking at someone’s face’ causes longer durations of facial expressions.

From the total of 45 facial expressions, only four occurred exclusively when facing another individual. These four types are morphologically very similar and mostly represent variations of the ‘mouth open’ facial expressions and thus could also be interpreted as the same expression performed with varying degrees of intensity. However, although these facial expressions were not context-specific as they occurred in more than one context, future studies are needed to investigate whether these variants of one facial expression serve different functions [43].

Furthermore, facial expressions when facing another individual occurred more frequently in social compared to non-social contexts. This shows that individuals direct facial expressions to the other individual more often when interacting with each other and that they influence the recipient’s behaviour in a way that they respond by using another facial expression (while other communicative behaviors were not considered in this study). Based on the current data, we can not rule out the potential explanation that activities in social contexts differ from those in non-social contexts, and as a consequence, they facilitate more frequent facial expressions and longer durations.

In order to examine whether directed facial expressions elicited a response of the recipient, we investigated whether facial expressions of one individual were immediately followed by another facial expression from their pair partner. We found that these consecutive facial expressions were significantly more common if the individual producing a facial expression was facing the recipient compared to events when the interacting individuals were not facing each other. This shows that recipients were influenced by the facial expressions of the other individual as indicated by their facial response.

Since we focused only on those consecutive facial expressions, which were different from the type of first facial expression produced, it is at least less likely that the response of the other individual was merely driven by facial mimicry i.e. the mechanism, which induces the same facial expression in others [e.g. 30]. The exclusion of facial mimicry instances can be considered as very conservative as they could potentially also include instances of purposeful communication instead of merely reflexive reactions to the sender’s initial facial expression. For example, it has been shown (at least in chimpanzees for non-human primates) that individuals can be selective as to which facial expression is mimicked or not [44]. This seems to support the conclusion that higher level cognitive processes are involved. However, to fully understand how hylobatids are using consecutive facial expressions and how this might interact with other more reflexive behaviours (such as mimics of other expressions) future research needs to be conducted.

To determine in more detail whether facial expressions are under purely voluntary control compared to reflexive production, experimental studies are necessary in order to maximally rule out other low-level explanations [e.g. 9]. Since we only considered facial expressions, but not gestures, body postures or vocalizations as possible reactions to an initial facial expression, we could have underestimated the reactions elicited by facial expressions. Therefore, future studies should apply a multimodal approach to narrow down the mechanisms underlying communicative expressive signals in non-human primates' social interactions.

Together our findings suggest that small apes have at least some control over the production of their facial expressions because they use them differentially depending on the recipient’s state of attention. This suggests that some gibbon facial expressions do indeed represent intentionally used signals. However, the adjustment to the recipient’s behaviour is just one of several criteria to identify instances of intentional communication [45]. Future research needs to consider additional markers of intentionality to fully conclude intentional use of facial expressions.

## Supporting Information

S1 Table Information about individuals and pair composition. (DOCX)

S2 Table List of facial expressions. Different types of facial expressions observed, number of occurrence, contexts and references to similar descriptions in the literature.
(DOCX)

S3 Table Summary of all facial expression events. Facial expression events for each individual (ID) categorized by context and facing (F) and non-facing (NF) in the whole data set. Explicit descriptions, which instances are excluded from the analyses, are marked. Grey marked categories were excluded from the analyses. Rationales for exclusion are provided in description below table.
(DOCX)

S4 Table Data file. This table includes all data points (facial expression events) which occurred given our criteria (see Methods). It contains information about the signalling individual (species, gender, individual of which pair) and information about the signal (AU combination, duration, context and whether individuals of the pair were facing each other or not).
(CSV)
