# Supplementary material for: Social Use of Facial Expressions in Hylobatids
Source: PLoS One. 2016 Mar 15;11(3):e0151733. doi: 10.1371/journal.pone.0151733 (PMC4792372; doi:10.1371/journal.pone.0151733)
Supplement: S1 Table — (DOCX) [file pone.0151733.s001.docx]

S1 Table: Information about individuals and pair composition.

| Pair | Individuals | Species | Birth | Sex | Zoo |
| --- | --- | --- | --- | --- | --- |
| **1** | Daniel | *Symphalangus syndactylus* | 26.05.1996 | m | Twycross |
|  | Tango | *Symphalangus syndactylus* | 27.03.1994 | f | Twycross |
| **2** | Kane | *Symphalangus syndactylus* | 02.11.1990 | m | Twycross |
|  | Sheena | *Symphalangus syndactylus* | 30.01.1991 | f | Twycross |
| **3** | Spike | *Symphalangus syndactylus* | 25.11.2000 | m | Twycross |
|  | Tarragona | *Symphalangus syndactylus* | 18.11.2000 | f | Twycross |
| **4** | Khmer | *Hylobates pileatus* | 28.11.1984 | m | Zurich |
|  | Willow | *Hylobates pileatus* | 06.05.1987 | f | Zurich |
| **5** | Iaman | *Hylobates pileatus* | 1959 | m | Zurich |
|  | Iba | *Hylobates pileatus* | 1974 | f | Zurich |
| **6** | Dan | *Nomascus gabriellae* | 1991 | m | Mulhouse |
|  | Chloe | *Nomascus gabriellae* | 06.01.1990 | f | Mulhouse |
| **7** | Dorian | *Nomascus siki* | 23.12.1989 | m | Mulhouse |
|  | Fanny | *Nomascus siki* | 13.06.1993 | f | Mulhouse |
| **8** | Bert | *Hylobates lar* | 01.05.1982 | m | Rheine |
|  | Lissy | *Hylobates lar* | ca. 1981 | f | Rheine |
