# Supplementary material for: Social Use of Facial Expressions in Hylobatids
Source: PLoS One. 2016 Mar 15;11(3):e0151733. doi: 10.1371/journal.pone.0151733 (PMC4792372; doi:10.1371/journal.pone.0151733)
Supplement: S2 Table — Different types of facial expressions observed, number of occurrence, contexts and references to similar descriptions in the literature. (DOCX) [file pone.0151733.s002.docx]

S2 Table: List of facial expressions. Different types of facial expressions observed, number of occurrence, contexts and references to similar descriptions in the literature.

| No | Facial expression  in ascending order of complexity (# of AUs/ADs)  [Number of occurrence] | Contexts** | OMF*** | Same/similar facial expressions in other non-human primates observed in the literature |
| --- | --- | --- | --- | --- |
| 1 | AU1+2 [12] | g, r, s |  |  |
| 2 | AU8 [1] | u |  |  |
| 3 | AU12 [1] | r |  |  |
| 4 | AU17 [4] | c, r, s |  |  |
| 5 | AU18 [11] | r, s, u |  |  |
| 6 | AU25 [14] | r, s, u | **+** |  |
| 7 | AU41 [11] | p, r, s, u |  |  |
| 8 | AUEye* [3] | a, r |  |  |
| 9 | AD500 [5] | n, r, s |  |  |
| 10 | AU1+2+AU18 [2] | r |  |  |
| 11 | AU10+AU25 [1] | r | **+** |  |
| 12 | AU16+AU25 [2] | r | **+** | Described as open-mouth play face in chimpanzees [3] used exclusively with laughter vocalization |
| 13 | AU25+AU26 [193] | a, g, n, p, r, s, u | **+** | Most subtle ‘open mouth’ facial expression [1,2] in orang-utans and siamangs; also described in [3] found as open-mouth play face with laughter and silent open-mouth face in chimpanzees |
| 14 | AU25+AU27 [52] | a, g, p, r, u | **+** | Intense ‘open mouth’ facial expression [1,2] in orang-utans and siamangs; also described in [3] found as open-mouth play face with laughter and silent open-mouth face in chimpanzees |
| 15 | AU41+AUEye [2] | r |  |  |
| 16 | AU7+AU25+AU26 [5] | r | **+** |  |
| 17 | AU8+AU25+AU26 [16] | g, r, s | **+** |  |
| 18 | AU10+AU25+AU26 [18] | r, s, u | **+** | Described in [3] as open-mouth face with laughter and silent open-mouth face in chimpanzees |
| 19 | AU10+AU25+AU27 [16] | a, p, r u | **+** | Described in [3] as open-mouth face with laughter and silent open-mouth face in chimpanzees |
| 20 | AU12+AU25+AU26 [6] | a, g, p, u | **+** | ‘Play face’ without teeth exposure in e.g. chimpanzees [4] and gorillas [5]; also described in [3] found as open-mouth play face with laughter and silent open-mouth face in chimpanzees |
| 21 | AU12+AU25+AU27 [6] | a, g, p, r | **+** | Intense ‘play face’ without teeth exposure e.g. in gorillas [5]; also described as open-mouth play face in chimpanzees [3] used exclusively without laughter vocalization |
| 22 | AU16+AU25+AU26 [57] | a, c, g, p, r, s, u | **+** | Described in [3] as open-mouth play face with laughter and silent open-mouth face in chimpanzees |
| 23 | AU16+AU25+AU27 [42] | a, p, r, s, u | **+** | Described in [3] as open-mouth play face with laughter and silent open-mouth face in chimpanzees |
| 24 | AU18+AU25+AU26 [2] | g, r, s | **+** |  |
| 25 | AU25+AU26+AD19 [5] | g, r | **+** |  |
| 26 | AU25+AU26+AD37 [435] | c, g, n, r, s, u | **+** |  |
| 27 | AU25+AU27+AD19 [7] | s | **+** |  |
| 28 | AU1+2+AU5+AU25+AU26 [1] | r | **+** |  |
| 29 | AU8+AU25+AU26+AD19 [1] | r | **+** |  |
| 30 | AU8+AU25+AU26+AD37 [38] | g, p, r, s, u | **+** |  |
| 31 | AU9+AU10+AU25+AU27 [2] | r | **+** |  |
| 32 | AU10+AU12+AU25+AU27 [2] | a | **+** | ‘Scream’ face in chimpanzees [4], sometimes AU16 (lower lip depressor) included; also described as open-mouth play face in chimpanzees [3] used exclusively with laughter vocalization |
| 33 | AU10+AU16+AU25+AU26 [16] | p, r, u | **+** | Described in [3] as open-mouth face with laughter and silent open-mouth face in chimpanzees |
| 34 | AU10+AU16+AU25+AU27 [80] | a, n, p, r, s, u | **+** | Described in [3] as open-mouth face with laughter and silent open-mouth play face in chimpanzees |
| 35 | AU12+AU16+AU25+AU26 [4] | a, p, u | **+** | Mild ‘Play face’ with teeth exposure (AU16=lower lip depressor), e.g. in gorillas [5]; also described in [3] as open-mouth play face with laughter and silent open-mouth face in chimpanzees |
| 36 | AU12+AU16+AU25+AU27 [5] | a, p, u | **+** | Intense ‘Play face’ with teeth exposure (AU16=lower lip depressor), e.g. in gorillas [5]; also described in [3] found as open-mouth play face with laughter and silent open-mouth face in chimpanzees |
| 37 | AU12+AU25+AU26+AD37 [1] | r | **+** |  |
| 38 | AU25+AU26+AUEye+AD37 [1] | r | **+** |  |
| 39 | AU25+AU26+AD37+AD500 [1] | u | **+** |  |
| 40 | AU16+AU25+AU26+AUEye [1] | a | **+** |  |
| 41 | AU1+2+AU10+AU16+AU25+AU27 [1] | r | **+** |  |
| 42 | AU9+AU10+AU16+AU25+AU27 [1] | a | **+** | Can be also compared to very intense ‘Play face’ in e.g. gorillas [5] before play biting (lower (AU16) and upper (AU10) lip and nose wrinkler (AU9) active) |
| 43 | AU10+AU12+AU16+AU25+AU26 [6] | p, r | **+** | Can be also compared to mild ‘Play face’ (see 35) before play biting (lower (AU16) and upper (AU10) lip active); also described in [3] as open-mouth play face with laughter and silent open-mouth face in chimpanzees |
| 44 | AU10+AU12+AU16+AU25+AU27 [13] | a, p, r, u | **+** | ‘Scream’ face in chimpanzees [4], sometimes without AU16; also described in [3] as open-mouth play face with laughter and silent open-mouth face in chimpanzees |
| 45 | AU10+AU12+AU16+AU25+AU27+  AUEye [2] | p, s | **+** | ‘Scream’ face (see 44) in chimpanzees with closed eyes |

(* AUEye resembles either AU43 (eye closure) or AU45 (eye blink), we did not differentiate between the two AUs here / ** Contexts (a=agonistic, g=grooming, s=self-grooming, n=nursing, p=play, r=resting, c=copulation, u=unclear / *** open mouth facial display).

References

1. Waller BM, Caeiro CC, Davila-Ross M. Orangutans modify facial displays depending on recipient attention. PeerJ. 2015;3: e827. doi:10.7717/peerj.827

2. Liebal K, Pika S, Tomasello M. Social communication in siamangs (Symphalangus syndactylus): use of gestures and facial expressions. Primates. 2004;45: 41–57. doi:10.1007/s10329-003-0063-7

3. Davila-Ross M, Jesus G, Osborne J, Bard K. Chimpanzees (Pan troglodytes) Produce the Same Types of “Laugh Faces” when They Emit Laughter and when They Are Silent. PLoS One. 2015;10: e0127337. doi:10.1371/journal.pone.0127337

4. Parr LA, Waller BM. Understanding chimpanzee facial expression: insights into the evolution of communication. Soc Cogn Affect Neurosci. 2006;1: 221–8. doi:10.1093/scan/nsl031

5. Waller BM, Cherry L. Facilitating play through communication: significance of teeth exposure in the gorilla play face. Am J Primatol. 2012;74: 157–64. doi:10.1002/ajp.21018
