# Supplementary material for: Social Use of Facial Expressions in Hylobatids
Source: PLoS One. 2016 Mar 15;11(3):e0151733. doi: 10.1371/journal.pone.0151733 (PMC4792372; doi:10.1371/journal.pone.0151733)
Supplement: S3 Table — Facial expression events for each individual (ID) categorized by context and facing (F) and non-facing (NF) in the whole data set. Explicit descriptions, which instances are excluded from the analyses, are marked. Grey marked categories were excluded from the analyses. Rationales for exclusion are provided in description below table. (DOCX) [file pone.0151733.s003.docx]

S3 Table: Summary of all facial expression events. Facial expression events for each individual (ID) categorized by context and facing (F) and non-facing (NF) in the whole data set. Explicit descriptions, which instances are excluded from the analyses, are marked. Grey marked categories were excluded from the analyses. Rationales for exclusion are provided in description below table.

|  | **Social context**  **F = Facing / NF: Non-facing**  **(Copulation and Nursing excluded from analyses*)** | | | | | **Non – social context** | | **Excluded**  **from**  **analyses** | **Total**  **A: in analyses** |
| --- | --- | --- | --- | --- | --- | --- | --- | --- | --- |
| ID | Agonism | Grooming | Self-Grooming | Copulation | Nursing | Play | Resting | Unclear |  |
| 1 | F: 20  NF: 8 | F: 3  NF: 26 | F: 1  NF: 25 | F: 0  NF: 2 | F: 0  NF: 1 | F: 31  NF: 50 | F: 9  NF: 79 ^(1)*^ | F: 14  NF: 50 ^(1)**^ | 319 ^(2)**^  **A: 252** |
| 2 | F: 20  NF: 10 | F: 2  NF: 13 | F: 1  NF: 4 | F: 0  NF: 3 | F: 2  NF: 8 | F: 19  NF: 16 | F: 3  NF: 21 | F: 16  NF: 41 | 179  **A: 109** |
| 3 | F: 1  NF: 2 | F: 2  NF: 5 | F: 0  NF: 12 ^(2)**^ | F: 0  NF: 2 | F: 0  NF: 0 | F: 3  NF: 2 | F: 4  NF: 33 ^(1)*^ | F: 6  NF: 13 | 85 ^(3)**^  **A: 64** |
| 4 | F: 4  NF: 3 | F: 2  NF: 14 | F: 2  NF: 15 | F: 0  NF: 1 | F: 0  NF: 7 ^(1)**^ | F: 7  NF: 11 | F: 3  NF: 47 | F: 14  NF: 20 | 150 ^(1)**^  **A: 108** |
| 5 | F: 5  NF: 1 | F: 1  NF: 3 | F: 0  NF: 0 | F: 0  NF: 0 | F: 0  NF: 0 | F: 1  NF: 4 | F: 2  NF: 33 | F: 9  NF: 16 | 75  **A: 50** |
| 6 | F: 2  NF: 0 | F: 5  NF: 10 | F: 0  NF: 1 | F: 0  NF: 0 | F: 0  NF: 0 | F: 0  NF: 0 | F: 3  NF: 42 | F: 9  NF: 6 | 78  **A: 63** |
| 7 | F: 0  NF: 0 | F: 0  NF: 0 | F: 0  NF: 0 | F: 0  NF: 0 | F: 0  NF: 0 | F: 0  NF: 0 | F: 1  NF: 22 | F: 0  NF: 0 | 23  **A: 23** |
| 8 | F: 0  NF: 0 | F: 1  NF: 1 | F: 0  NF: 0 | F: 0  NF: 0 | F: 0  NF: 0 | F: 0  NF: 0 | F: 2  NF: 11 | F: 1  NF: 0 | 16  **A: 15** |
| 9 | F: 0  NF: 0 | F: 0  NF: 3 | F: 0  NF: 6 | F: 0  NF: 0 | F: 0  NF: 0 | F: 0  NF: 0 | F: 0  NF: 32 | F: 1  NF: 10 | 52  **A: 41** |
| 10 | F: 0  NF: 0 | F: 0  NF: 0 | F: 0  NF: 0 | F: 0  NF: 0 | F: 0  NF: 1 | F: 0  NF: 0 | F: 1  NF: 23 | F: 0  NF: 2 | 27  **A: 24** |
| 11 | F: 0  NF: 0 | F: 0  NF: 0 | F: 0  NF: 0 | F: 0  NF: 0 | F: 0  NF: 0 | F: 0  NF: 0 | F: 3  NF: 7 | F: 0  NF: 2 | 12  **A: 10** |
| 12 | F: 0  NF: 0 | F: 0  NF: 2 | F: 0  NF: 1 | F: 0  NF: 0 | F: 0  NF: 0 | F: 0  NF: 0 | F: 0  NF: 7 | F: 0  NF: 1 | 11  **A: 10** |
| 13 | F: 0  NF: 0 | F: 0  NF: 0 | F: 0  NF: 0 | F: 0  NF: 0 | F: 0  NF: 0 | F: 0  NF: 0 | F: 0  NF: 0 | F: 1  NF: 1 | 2  **A: 0** |
| 14 | F: 0  NF: 0 | F: 1  NF: 0 | F: 0  NF: 0 | F: 0  NF: 0 | F: 0  NF: 0 | F: 0  NF: 0 | F: 1  NF: 0 | F: 0  NF: 0 | 2  **A: 2** |
| 15 | F: 0  NF: 0 | F: 0  NF: 13 | F: 0  NF: 7 | F: 0  NF: 0 | F: 0  NF: 0 | F: 0  NF: 0 | F: 2  NF: 12 | F: 3  NF: 3 | 40  **A: 34** |
| 16 | F: 0  NF: 0 | F: 2  NF: 4 | F: 2  NF: 2 | F: 0  NF: 0 | F: 0  NF: 0 | F: 0  NF: 0 | F: 1  NF: 10 | F: 1  NF: 7 | 29  **A: 21** |
|  | 76 | 113 | 79 ^(2)**^ | **27** ^(1)**^ | | 144 | 414 ^(2)*^ | **247** ^(1)**^ | 1100 ^(6)**^ |
|  | **Social: 268** | | |  | | **Non-social: 558** | |  | **A: 826** |
| Total: 1106 – 27 (excluded from Copulation & Nursing) – 6 (excluded from AD500) = 1073  Total: 1073 – 247 (excluded from Unclear) = 826 | | | | | | | | |  |

[* We excluded Copulation and Nursing from the analyses. The rational behind this exclusion is that Copulation excludes instances of facing since the way gibbon species copulate is from mounting the other from behind. The probability that individuals are facing each other is low. Facial expressions during Nursing are most likely directed to the infant and not to the pair partner. Also, not all individuals had infants at the time of data collection.

** We excluded all facial expressions containing AD500 (= throat sack inflation) since only siamangs have a throat sack, not the other species.]
